# Effects of oxidative stress on liver, brain and spinal cord of rats using L-NAME and treated with hydroxyurea. A model of sickle cell complication^1^

**DOI:** 10.1590/s0102-865020200030000001

**Published:** 2020-05-08

**Authors:** Abilio Torres dos Santos, Iandara Schettert Silva, Maria Lucia Ivo, Camila Tozaki Rodrigues, Eduardo Benedetti Parisotto, Rondon Tosta Ramalho, Geanlucas Mendes Monteiro

**Affiliations:** I Fellow Master degree, Postgraduate Program in Health and Development in the Midwest Region, Universidade Federal do Mato Grosso do Sul (UFMS), Campo Grande-MS, Brazil. Conception and design of the study; technical procedures; acquisition, interpretation and analysis of data; manuscript writing.; II PhD, Associate Professor, Postgraduate Program in Health and Development in the Midwest Region, Laboratory of Experimental Carcinogenicity, UFMS, Campo Grande-MS, Brazil. Conception and design of the study, interpretation of data, manuscript writing, critical revision, final approval.; III Full Professor, Postgraduate Program in Health and Development in the Midwest Region, Coordinator of the Epidemiological Studies Laboratory, UFMS, Campo Grande-MS, Brazil. Conception and design of the study, interpretation of data, manuscript writing, critical revision, final approval.; IV Specialist Nurse in Obstetrics, School of Nursing, Universidade de São Paulo (USP), Brazil. Design of the study, technical procedures.; V PhD, Assistant Professor, Faculty of Pharmaceutical Sciences, Food and Nutrition, UFMS, Campo Grande-MS, Brazil. Interpretation of data, manuscript writing, critical revision, final approval.; VI PhD, Full Professor, Postgraduate Program in Health and Development in the Midwest Region, Laboratory of Experimental Carcinogenicity, UFMS, Campo Grande-MS, Brazil. Interpretation of data, manuscript writing, critical revision, final approval.; 7 Fellow Master degree, Postgraduate Program in Health and Development in the Midwest Region, UFMS, Campo Grande-MS, Brazil. Conception and design of the study, technical procedure.

**Keywords:** Anemia, Sickle Cell, Oxidative Stress, Hydroxyurea, NG-Nitroarginine Methyl Ester, Rats

## Abstract

**Purpose:**

To analyze the serum levels of nitric oxide and correlate them with the levels of thiobarbituric acid reactive substances (TBARS) in liver, brain and spinal cord of animals using L-NAME and treated with hydroxyurea.

**Methods:**

Eighteen male albino Wistar rats were divided into three groups. N^G^-nitro-L-arginine methyl ester (L-NAME) was intraperitoneally administered to induce oxidative stress. TBARS and plasma nitric oxide levels were analyzed in all groups. Histopathology of the liver and vascular tissue was performed.

**Results:**

Statistically significant differences were seen in liver, brain and spinal cord TBARS levels.

**Conclusions:**

Following the use of L-NAME, hepatic tissue increased the number of Kupffer cells as oxidative stress and inflammatory response increased. The use of L-NAME caused an increase in lipid peroxidation products and, consequently, in oxidative stress in animals. Hydroxyurea doses of 35 mg / kg / day reduced TBARS values in liver, brain and spinal cord.

## Introduction

Sickle cell disease (SCD) is a term associated with the group of hemoglobinopathies, characterized by the mutation of chromosome 11 that causes the change of a nitrogen base pair (adenine → thymine) in the sixth position of the beta globin chain; consequently, this change causes a replacement of glutamic acid with a valine, thus producing hemoglobin S (HbS)^1^.

Hemoglobin S may occur in the homozygous SS form, called sickle cell anemia, or in association with other structural variants SC, SD as well as in association with β, Sβ + thalassemia, Sβ0 thalassemia. Also in AS heterozygosis or sickle cell trait, both asymptomatic^2^.

The punctual mutation that occurs with this process leads to frequent hemoglobin polymerization, causing cellular deformation, so that the red blood cell acquires an elongated shape of “sickle”. By adopting this format, several events occur in blood cells, such as vaso-occlusion, chronic hemolysis and consequent episodes of pain. The mechanisms of vaso-occlusion are not yet fully understood^3^.

Chronic hemolysis triggers extravasation of hemoglobin in plasma, transforming nitric oxide into inactive nitrate (Reactive Oxygen Species - ROS). This event allows the release of arginase, an enzyme capable of hydrolyzing L-arginine, a necessary component for nitric oxide (NO) synthesis, making this molecule reduce^4^.

In sickle cell anemia, the reduction in NO synthesis causes several changes: vascular inflammation, increased free radicals, stroke, pulmonary hypertension, and liver disease; such situations can be observed as a result of the sickling process and consequent microcirculation obstruction^5^.

This set of changes intensifies a continuous physiological process that is characterized by the generation of free radicals. However, if this production is excessive, it can generate oxidative stress leading to oxidative damage. Oxidative stress occurs because of the imbalance between oxidizing agents and antioxidants. Its chronicity is associated with the development of several diseases: cardiovascular, carcinogenic and neurodegenerative ones^6^.

Oxidative stress leads to the formation of specific metabolites that can be identified and quantified by direct or indirect methods. These markers originate from lipoperoxidation, protein oxidation and deoxyribonucleic acid (DNA). An indirect method of performing this analysis is by measuring malonaldehyde (MDA), a major lipoperoxidation product, that can be measured by thiobarbituric acid reactive substances (TBARS). These analyses can be performed on fluids, tissues and blood^7^.

The main objective of the treatment of sickle cell anemia is the induction of fetal hemoglobin; moreover, it has beneficial factors such as induction of endothelial nitric oxide synthesis and consequent vasodilation^8^. In 1998, Hydroxyurea was approved by the Food and Drug Administration (FDA) for the treatment of sickle cell anemia in adults and, in 2007, for children and adults by the European Medicines Agency (EMeA)^9^.

However, in addressing vascular inflammation in sickle cell anemia in a model of analogous complications of sickle cell anemia in animals after the use of L-NAME, it was possible to observe systemic changes in some organs caused by increased oxidative stress and decreased nitric oxide. Considering these aspects, the question was: “what is the relationship between L-NAME and the reduced nitric oxide as to other organs likely to be affected in the model of sickle cell anemia analogous complications?”

In view of the above, it was necessary to analyze serum nitric oxide levels and to relate them to the liver, brain and spinal cord TBA levels of animals using L-NAME and treated with Hydroxyurea.

## Methods

All procedures and protocols were followed as approved by the Ethics and Animal Use Committee / CEUA / UFMS N. 646/2014.

For the experimental design, 22 male Wistar albino rats (*Rattus norvegicus*) were used in 2015. The animals were taken from the Central *Vivarium*, Universidade Federal do Mato Grosso do Sul (UFMS) in Campo Grande, MS.

Inclusion criteria were: animals weighing 250 grams at 10 weeks of age. Rats outside this weight and age range were excluded.

The allocated animals were kept in the same vivarium, in collective cages, for a maximum of 03 animals per unit, under 12-hour light and dark cycles maintained artificially, temperature controlled (22-25°C) and with feed and water supplied *ad libitum.*

### Experimental groups

#### Treatments and procedures

The drug used during the experiment was N^G^-nitro-L-arginine methyl ester (L-NAME) supplied by Sigma-Aldrich^®^. L-NAME is an odorless white powder with a molecular weight of 269.69, a compound with 50 mg / ml water solubility. Its molecular formula equals C_7_H_15_N_5_O_4_•HCl. After diluting 1g of the compound, a total volume of 20 ml of the drug was obtained, that was stored throughout the experiment at a temperature between 0°C and −20°C.

HU was also used, that is a water-soluble, hygroscopic and odorless powder. Its molecular formula is CH_4_N_2_O_2_. Each capsule contains a total of 500 mg HU, and the recommended starting doses for treatment is 10-20 mg/kg/day and the maximum doses, 25-35 mg/kg/day.

#### Experimental design

In all, 22 animals were used. Initially a pilot study was conducted with four animals distributed as follows:

**Pilot Study (Control):** included two rats that received no dose of the drug;

**Pilot L-NAME:** included one rat receiving 25 mg/kg/day of L-NAME administered intraperitoneally for three days;

**Pilot L-NAME + HU:** included one rat receiving 50 mg/kg/day of L-NAME administered intraperitoneally for three days and, at the end, a 35 mg/kg oral dose by gavage.

After the three-day period, euthanasia was performed to confirm anatomo-pathological changes with the aim of characterizing a pharmacological pattern and continuing the study. The animals participating in the pilot study were discarded from the final sample of research.

After the pilot study, 18 animals were distributed in three experimental groups:

**Group Control:** six rats without L-NAME;

**Group L-NAME:** six rats receiving 25 mg/kg/day of L-NAME administered intraperitoneally for five days;

**Group L-NAME + HU:** six rats receiving 25 mg/kg/day L-NAME administered intraperitoneally for 10 days and, at the end, each animal receiving a 35 mg/kg oral dose by gavage.

Intraperitoneal L-NAME 0.5 ml was administered daily in both experimental groups according to the animal’s weight^10^.

HU doses were administered orally by gavage in a variable volume according to the weight of each animal using the maximum dose of 35 mg/kg; the dose was chosen according to the maximum dose used in the treatment of adult subjects. Exposure time was 24 hours.

Euthanasia occurred on the last day of the experiment with animals anesthetized with ketamine (Dopalen^®^ 100mg/kg) and xylazine (Anasedan^®^ 10mg/kg); Both solutions were administered intraperitoneally (i.p.). Soon afterwards, intracardiac puncture exsanguination was performed to complete euthanasia. Once anesthetized, the animals underwent total laparotomy and total thoracotomy to collect liver and great vessels for further histopathology.

### Histology

#### Preparation of histological sections

For the preparation of the slides, the liver and the great vessels were cleaved in transverse sections and underwent the standard histological technique: dehydration (70 °GL to 100 °GL ethanol), xylol I and II diaphanization, impregnation and inclusion in Histosec (Merck, Darmstadt, Germany). Then, 5 μm thick histological sections were made in microtome. For the examination of cellular architecture, the sections were stained by Hematoxylin-Eosin (HE) histological techniques for histological description of the organ. After the stains were applied, a coverslip and Entellan were overlaid to finish the preparation of slides^15^.

#### Observations and photomicrographs

All slides were observed and photographed using the Leica DM 5500 B microscope and Leica Application Suite Software - Version 3.8.0 (Build: 878), located and licensed by the Institute of Biology - INBIO, Federal University of Mato Grosso do Sul (UFMS).

#### Slide analysis

Computer analysis was performed using the ImageJ^®^, a specific computer program for image editing and formatting (National Institutes of Health, USA).

### Statistical analysis

After performing the NO measurement of the samples, the one-way ANOVA analysis method was used; for statistical analysis, the statistical software SigmaPlot version 12.5.

## Results

**Figure 1:**
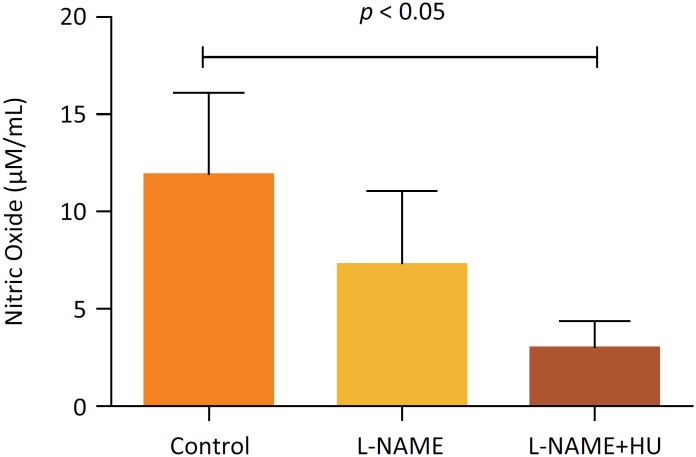
Concentration of NO in Groups Control, L-NAME and L-NAME+HU.

## Discussion

### Nitric oxide bioavailability

In the control group, the maximum available plasma serum nitric oxide level can be observed. Compared with the groups using L-NAME and L-NAME + hydroxyurea, a significant reduction was seen in the bioavailability of serum nitric oxide. Figure 1 shows the descriptive analysis of nitrite values in the different groups studied (p <0.05). It is widely known that the use of L-NAME is capable of inhibiting endogenous NO production synthesis^16^. Following the reduction of NO, the administration of HU has shown to be able to modulate the physiology of red blood cells and, consequently, of nitric oxide synthase, causing its higher bioavailability^17^. Regarding the bioavailability of nitric oxide, there was no expected increase. It should be noted that exposure to hydroxyurea was observed after 24 hours.

Figure 3 shows the analysis of histological sections of the great cardiac vessels in the respective groups. Even though these groups did not present statistical difference in the TBA analysis, a histological change was seen in Group L-NAME and, consecutively, with the treatment with hydroxyurea. In Group L-NAME, an increase in the middle layer was observed.

**Figure 2:**
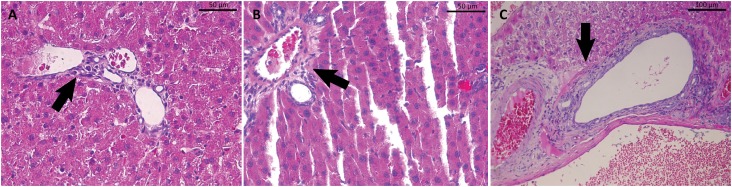
Histopathological slide of liver of rats of the three groups showing an increased Kupffer cell.: **(A)** Liver tissue in Group Control; **(B)** Liver tissue in Group L-NAME; **(C)** Liver tissue in Group L-NAME+HU (x400 magnification).

**Figure 3:**
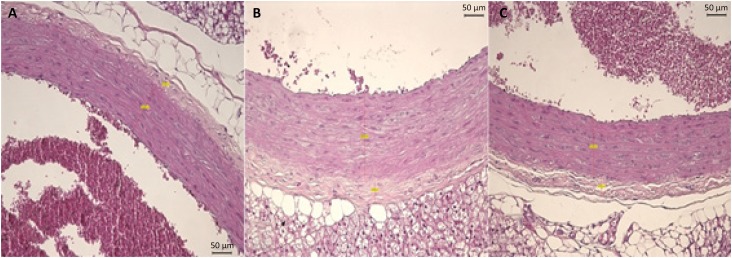
Histological sections of the great cardiac vessels of the three groups. **(A)** Great cardiac vessels in Group Control; **(B)** Great cardiac vessels in Group L-NAME; **(C)** Great cardiac vessels in Group L-NAME+HU (x400 magnification).

### Oxidative stress and the impact of endothelial nitric oxide blockade

Table 1 presents the descriptive analysis of the values of lipid peroxidation products, verified by TBARS dosage in three different tissues: liver, brain and spinal cord. The descriptive analysis of the values of lipid peroxidation product was verified by TBARS at three different times: control group, after administration of L-NAME for five days, and after the administration of L-NAME for 10 days plus a dose of 35 mg/kg/day of hydroxyurea.

**Table 1:** Comparison among groups according to mean values of TBARS in liver, spinal cord and brain.

Group	Liver	Spinal cord	Brain	p
Control	17019.55	439.03	515.73	0.001
L-NAME	39561.83	3330.99	4290.95	
HU	12232.27	3154.08	3809.28	

At the first column, the mean values of liver tissue TBARS were analyzed, and a significant difference between the groups was observed. The mean of Group L-NAME (39,561.83) was twice as high as the Group Control (17,019.55), with a restoration of TBARS content after the use of HU (12,232.27), which may suggest restoration of peroxidation. In the studies by Selamoglu *et al*.^18^, after injecting the dose of L-NAME (40 mg/kg) for a period of two weeks, increased TBA was evident, resulting in higher levels of oxidative stress. The authors used L-NAME as a therapeutic measure for the treatment of liver damage, but did not observe improvement because of vascular and systemic oxidative stress.

Analyzing the oxidative stress markers of liver tissue in rats that used L-NAME for five days i.p., a 232% increase in TBARS was observed when compared to the values of Control Group. These data corroborate those found by Pedrosa *et al*.^19^, who evaluated the neutrophil-produced TBARS of patients with sickle cell disease, showing an increase of about 360% of this marker. This increase indicates minimal production of antioxidant agents and increased oxygen and nitrogen ER, exacerbating vascular damage. Figure 2 shows the increase of Kupffer cells around the vessel as oxidative stress increased.

At the second column, the tissue collected from the spinal cord was analyzed. A significant difference was seen between the mean values of TBARS obtained by the groups. The mean values of medullary TBARS obtained in Group L-NAME (3,330.99) were higher than those of Control Group (439.03); however, there was a restoration of lipoperoxidation compared with the group with HU (3,154.08). In the study by Woźniak *et al*.^20^, it was observed that medullary damage increased oxidative stress and lipid peroxidation levels. In addition, erythroid levels were shown to be higher.

At the third column, the brain TBARS values were analyzed. There was a significant difference between the means of TBARS obtained by the groups. Group L-NAME (4290.95) was higher than ControlGroup (515.73); however, the group that received the HU dose (3809.28) responded by restoring lipid peroxidation values. Because of the high lipid concentration, the brain undergoes intense lipid peroxidation, so that cell membrane unsaturated lipids react with the reactive species, altering membrane permeability and damaging proteins^20^. The high TBA values found were observed in the study and, even after the administration of HU, a low reduction of TBA values was observed.

Regarding the limitation of the study, the brain histology or myelography was not performed as material resources were insufficient, which, however, did not prevent the study objectives from being achieved.

## Conclusions

By histopathological assays, after the use of L-NAME, liver tissue showed an increase in the number of Kupffer cells as oxidative stress and inflammatory response rose.

Under the same analysis, Group L-NAME presents thickening of the endothelial middle layer whereas in Group L-NAME plus HU, a reduction was observed in the measurement of the endothelial middle layer because of the relaxation of great vessels.

The use of L-NAME causes an increase in lipid peroxidation products and, consequently, oxidative stress in animals.

Hydroxyurea dose of 35 mg/kg/day reduces the value of oxidative stress in the liver, brain and spinal cord.
